# An Anti-Disturbance Resilience Enhanced Algorithm for UAV 3D Route Planning

**DOI:** 10.3390/s22062151

**Published:** 2022-03-10

**Authors:** Zhining Xu, Long Zhang, Xiaoshan Ma, Yang Liu, Lin Yang, Feng Yang

**Affiliations:** 1 National Key Laboratory of Science and Technology on Information System Security, Systems Engineering Institute, Academy of Military Science, Beijing 100141, China; xznfep@126.com (Z.X.); jkwxsliuyang@outlook.com (Y.L.); yfzy1221@163.com (F.Y.); 2 Postgraduate College, Air Force Engineering University, Xi’an 710043, China; maxiaoshan@buaa.edu.cn

**Keywords:** resilience, risk assessment, three-dimensional route planning, A* algorithm, artificial potential fields algorithm

## Abstract

Considering that the actual operating environment of UAV is complex and easily disturbed by the space environment of urban buildings, the RoutE Planning Algorithm of Resilience Enhancement (REPARE) for UAV 3D route planning based on the A* algorithm and artificial potential fields algorithm is carried out in a targeted manner. First of all, in order to ensure the safety of the UAV design, we focus on the capabilities of the UAV body and build a risk identification, assessment, and modeling method such that the mission control parameters of the UAV can be determined. Then, the three-dimensional route planning algorithm based on the artificial potential fields algorithm is used to ensure the safe operation of the UAV online and in real time. At the same time, by adjusting the discriminant coefficient of potential risks in real time to deal with time-varying random disturbance encountered by the UAV, the resilience of the UAV 3D flight route planning can be improved. Finally, the effectiveness of the algorithm is verified by the simulation. The simulation results show that the REPARE algorithm can effectively solve the traditional route planning algorithm’s insufficiency in anti-disturbance. It is safer than a traditional A* route planning algorithm, and its running time is shorter than that of the traditional artificial potential field route planning algorithm. It solves the problems of local optimization, enhances the UAV’s ability to tolerate general uncertain disturbances, and eventually improves resilience of the system.

## 1. Introduction

Rotor UAVs have the advantages of low-state coupling and flexibility in space attitude, which greatly improves the work efficiency of the platform; thus, they are favored by practitioners of high-altitude operations. Human-in-the-loop UAV systems have certain capacity limitations. In some disturbed scenarios, the mis-operation of human pilots or communication delays will cause the rotor UAV to make a wrong flight route, which is obviously unreliable.

To solve the problems of autonomous anti-disturbance, obstacle avoidance and planning of UAVs, a variety of UAV-assisted algorithms for specific needs have been developed. Traditional UAV autonomous route planning algorithms mainly include the A* algorithm [1,2,3], ant colony algorithm [4,5,6,7], simulated annealing algorithm [8,9,10,11], Particle Swarm algorithm [12,13,14], artificial potential fields algorithm [15,16,17,18,19], etc. The A* algorithm, represented by a small amount of calculations and fast speed, has difficulties ensuring the safe operation of the UAV in an environment involving disturbance. The artificial potential fields algorithm which is safe and controllable needs to calculate too many repulsion fields in multiple complex obstacle scenes, and the calculation is complicated. When rotor UAVs perform actual flight tasks among complex urban buildings, they are likely to face sudden strong winds, showers, etc. The displacement deviation caused by these external disturbances to the UAV makes the UAV deviate from the mission route. The anti-disturbance performance of the traditional route planning algorithm is not strong, and it will deviate from the expected route in the disturbance scenario, and it may collide with obstacles. Once a rotor UAV collides, it often means serious consequences such as damage to the body and an uncontrolled crash. The safety control of UAVs has attracted extensive attention, and it has become necessary to study a route planning algorithm that considers both safety and efficiency.

In order to realize the smooth operation of the UAV in the disturbance field, the traditional artificial potential fields algorithm and some common improved A* algorithms can make the UAV keep an extra safe distance from the obstacle in the disturbance scene. Even if the UAV is disturbed and shows a tendency to deviate from the route, it can also be returned to the route. When these algorithms are applied to the situations in which the disturbance intensity changes at any time and the change intensity span is large, they may cause insufficient or excessive correction, which will lead to security risks.

On the basis of improving the A* algorithm and the artificial potential fields algorithm, this paper proposes the REPARE algorithm based on risk assessment, such that the UAV can operate independently and safely. In the face of disturbance, UAVs identify risks, assess the risks, counter the disturbance, and correct the route. When facing obstacles, they dynamically avoid obstacles and plan routes. This fully reflects the UAV’s tolerance to unstable factors, giving the system a certain resilience.

In the second section, this paper will introduce the UAV resilience and the process of determining the UAV resilience implementation through disturbance analysis, risk assessment and dynamic route planning. The third section presents the simulation results and comparative validation of the REPARE algorithm.

The main significances of this paper are as follows:

1. This paper analyzes the common disturbance scenarios of the rotor UAV and the force after the disturbance, and it realizes the resilience of the UAV’s navigation route through the parallel guarantee of two sets of mechanisms.

2. The UAV risk identification, risk assessment and risk prediction models are designed through the mechanism of determining a reasonable safety range, such that the UAV can have a preliminary evaluation of the task risk and difficulty before flying and can correct the resilience coefficient in advance.

3. Using the method of machine learning (XGBoost), the discrete degree of risk distribution of the UAV’s route planning in the disturbed scene is predicted.

4. By adjusting the potential risk compensation penalty weight coefficient and by dynamically adjusting the obstacle avoidance parameters, the UAV’s real-time variable strategy route planning is realized.

5. Through the online real-time monitoring mechanism, the status of the UAV is monitored in real time. After approaching the obstacle for a certain distance, the obstacle avoidance module is turned on in time to maintain a sufficient safety distance. When encountering unforeseen risk factors, there are still possibilities for readjustment, such that the navigation route of the UAV is safer.

6. Through the Monte Carlo simulation, the resilience of RoutE Planning Algorithm of Resilience Enhancement (REPARE) is verified.

## 2. Resilience Enhancement Design for UAV 3D Route Planning

### 2.1. UAV Resilience

UAV resilience [20,21] refers to the ability of the UAV auxiliary control system to withstand, absorb, recover and improve in the face of external disturbances. When disturbance occurs, the safe operation of the system is maintained by sacrificing part of the operating efficiency and enhancing the safety capacity. For example, the UAV reduces the maximum speed and the pitch angle. When the disturbance ends, the system returns to its pre-accident performance. If the UAV auxiliary control system can meet the above description, it is said that the UAV auxiliary control system has resilience, which is referred to as UAV resilience and which enables the UAV to take into account safety and efficiency while ensuring the operation of the mission.

#### 2.1.1. Ideas for the Realization of UAV Resilience

For an ideal scenario without disturbance, UAVs only need to fly in the order of pre-set mission points to perform tasks. In common disturbance scenarios, pre-existing route planning algorithms can achieve a certain degree of anti-disturbance. Under some strong disturbance and strong loads, the risk may exceed the safety range of the UAV. According to the disturbance intensity of external factors, the risk is quantified according to the classification, which is beneficial to further research and optimization of the UAV resilience design.

**Figure 1:**
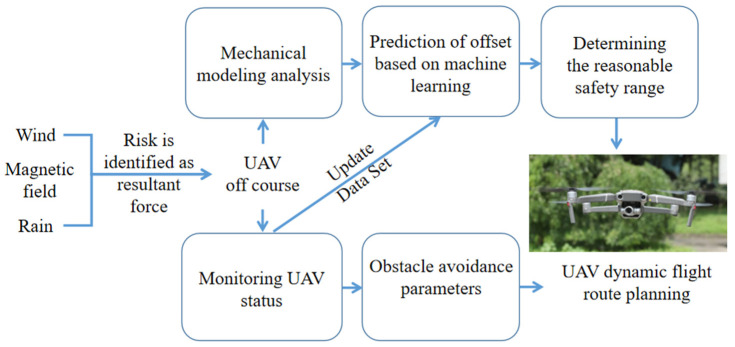
Block diagram of 3D route planning resilience enhancement design.

Figure 1 is the design block diagram of the resilience enhancement design of 3D route planning when the UAV is subjected to external disturbances such as wind, a magnetic field and rain.

As is shown in Figure 1, when the disturbance occurs, the UAV identifies potential security risks and uses the UAV’s two-layer security protection mechanism; that is, the UAV refers to its own physical entity performance to determine a reasonable security range mechanism and the UAV online real-time monitoring mechanism. The UAV describes the safety boundary according to the mechanism of determining a reasonable safety range, then dynamically adjusts the UAV route planning and obstacle avoidance parameters according to the online real-time monitoring mechanism, and selects the appropriate route resilience planning strategy for different levels of disturbance; thus, the resilience of the UAV is enhanced.

#### 2.1.2. UAV Risk Identification to Determine a Mechanism of Determining the Reasonable Safety Range

The mechanism of determining the reasonable safety range of the UAV (safety range insurance) is based on the UAV’s own physical entity performance, such as: maximum ascent speed, maximum horizontal flight speed, maximum tilt angle, maximum wind resistance level, maximum load and other constraints. Before the UAV is put into operation, the test flight status information of the UAV under various experimental conditions is collected, and the quantitative modeling and analysis of the degree of disturbance of the UAV is carried out. The offset is used as a labeled dataset for training, and an analysis and correction model of the offset after the UAV is disturbed is obtained. The model is finally deployed on the UAV, and the difficulty of the task is predicted and judged according to the task scene analysis so as to realize the UAV resilience route planning of each task.

UAV risk identification is the real-time input of the UAV based on prior inputs, such as load, wind speed, rainfall and various sensors. Based on the comprehensive judgments of prior data and real-time data, the REPARE algorithm pre-sets the flight strategy for this mission and completes the flight preparations according to the judgments of risk assessment.

#### 2.1.3. Dynamic Track Planning of Online Real-Time Monitoring Mechanism

When the system is running, the online real-time monitoring monitors the operating status of the system online to ensure the real-time security of the system. It is mainly composed of a basic controller, advanced controller and a monitor. The basic controller can meet the basic control needs (baseline), and the advanced controller is better than the basic controller in the security performance of the control object, but it also pays a certain price of efficiency (trade-off). The monitor monitors the running status and selects the appropriate controller for adaptation.

UAV dynamic route planning is a real-time monitoring of the disturbed field by the UAV according to its own environment and sensors, and it adjusts the working intensity of the obstacle avoidance module according to the needs for anti-disturbance.

### 2.2. Risk Assessment of Mechanisms for Determining Reasonable Security Scope

UAVs face different disturbances in different mission scenarios such as wind, rain, sand, magnetic fields, etc. Its essence can be summarized as displacement offset disturbance and load disturbance. In this paper, the application of the REPARE algorithm is illustrated by taking the UAV flying in a windy and rainy building group, as an example.

#### 2.2.1. Disturbance Analysis of UAV in Wind and Rain

The maximum ascent speed of the UAV is limited by the power of the engine. In the Cartesian coordinate system, the ascent is in the same direction as the positive z-axis, setting maximum lift as $F_{zMax}$. All symbols and definitions can be found in Table A1 in the Appendix A.

Considering the disturbance in the direction of gravity, for example, when the UAV is flying in rainy days, the pressure $\;P_{r}\;$ will be generated when the raindrops fall on the UAV’s body. The effective area of the UAV’s body which can be hit by the rain is $S_{r}$, and the falling speed of the raindrops is $V_{r}$. The raindrops rebound after falling on the fuselage of the UAV, the momentum loss of rainwater is $\eta_{r}$. Within unit time t, the amount of precipitation is $H_{r}$, and the density of the rainwater is $\rho_{r}.$ In the end, the vertical force of the rain when the UAV is flying in the rain is $F_{r}$, and the resilience coefficient fulfilling the flight safety of the UAV is $\delta_{r}.$

(1) $$F_{r}=P_{r}S_{r}=\frac{H_{r}\rho_{r}V_{r}\left(1-\eta_{r}\right)}{t}$$

Setting the UAV self-weight as $G_{uav}$, UAV load as $G_{s}$, and vertical disturbance force as $F_{r}$:

(2) $$F_{zMax}>\delta_{r}\left(G_{uav}+G_{s}\right)+F_{r}$$

According to the weather forecast, the forecasted average wind speed $\vec{V_{\mu F}}$ and maximum wind speed $\vec{V_{MaxF}}\;$ of the day can generally be known as prior knowledge. The average wind speed per unit time $t_{w}$ measured by the UAV sensor in the current flight environment is $\vec{V_{\mu S}}$. According to the comprehensive consideration of the predicted wind speed and the measured wind speed, the larger value is taken as the basis for the instantaneous risk assessment.

(3) $$V_{res}=\mathrm{Max}\left(\left|\vec{V_{\mu F}}\right|,\left|\vec{V_{\mu S}}\right|\right)$$

The maximum speed that the UAV can reach in a no-wind state is $\vec{V_{uav}}$. When the UAV’s flight speed relative to the ground $\vec{v}\;$ meets the mission required speed $V_{d}$, the equation is as follows:

(4) $$\vec{V_{d}}\leq \vec{v}=\vec{V_{wMax}}+\vec{V_{uav}}$$

The force in the horizontal direction of the UAV according to the air resistance formula is calculated:

(5) $$F_{hmax}=\frac{1}{2}C\rho_{a}S_{w}\vec{V_{d}}$$

C  is the air resistance coefficient, $\rho_{a}\;$ is the air density, and $S_{w}\;$ is the equivalent area of the UAV in contact with the wind.

Through the calculation, the disturbance force perpendicular to the ground $F_{zMax}$ and the disturbance force in the horizontal direction $F_{hmax}$ can be obtained, and the data records of previous missions can be collected for risk prediction.

#### 2.2.2. Disturbance Classification Model Based on Gaussian Distribution

According to the current scene of the UAV performing the task, the obstacle avoidance parameters will be set according to the prior data prediction, and the obstacle avoidance parameters at this time are macro parameters. When the UAV hovers in the air, the displacement difference per unit time is ΔS, and the disturbance force calculated in real time is F. After a large number of repeated experiments, the disturbance force F can be simulated to approximately fulfill the Gaussian distribution.

Let n(x) denote the probability density function of the one-dimensional shift  x under the σ level of risk and μ level of disturbance.

(6) $$n\left(x\right)=\frac{1}{\sqrt{2\pi }\sigma }\exp\left(-\frac{{\left(x-\mu \right)}^{2}}{2\sigma^{2}}\right)$$

**Figure 2:**
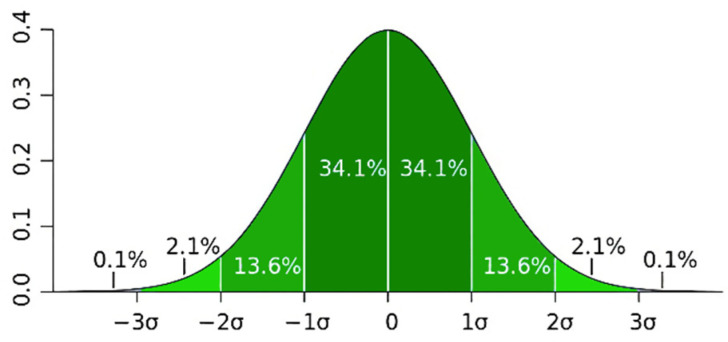
The interval that fulfils the Pauta (3σ) criterion.

As shown in Figure 2, according to the Pauta criterion, the results of the random test were standardized to obtain the standard deviation σ. Generally, only if the random result is within three standard deviation 3σ offsets, that is, within the probability interval of 99.7%, we consider that the result of the random experiment contains only random errors and no gross errors.

In the research on the universal requirements of UAV resilience, as long as the offset of the UAV after disturbance is within 3σ (the green filled part), we consider it to meet the resilience requirements.

**Figure 3:**
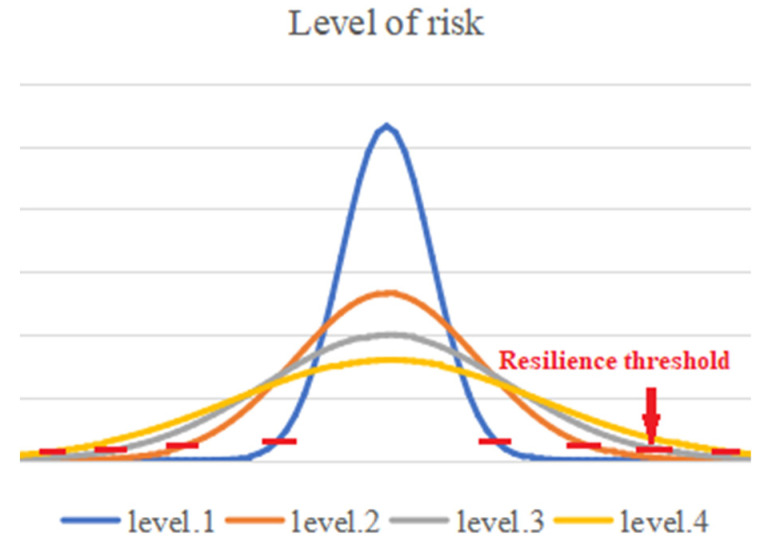
Schematic diagram of resilience thresholds for different risk levels.

Among them, according to different σ risk levels, an image is drawn for the offset difference per unit time ΔS, which is shown in Figure 3. When more than 99.7% of the probability of the disturbance force is within the interval, it can be considered safe. We can see from the safety discriminant point that different risk levels have different offsets to fulfil safety.

#### 2.2.3. Disturbance Risk Prediction

**Figure 4:**
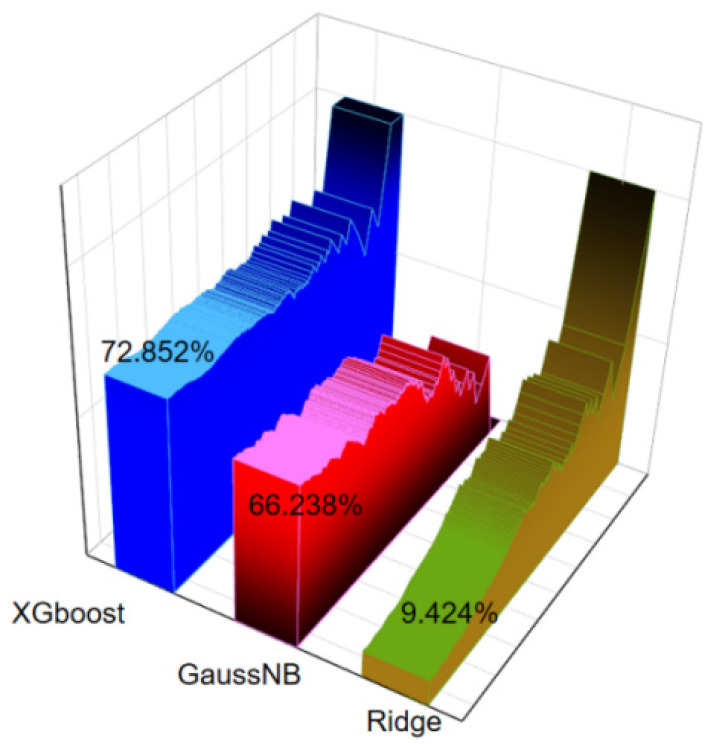
Disturbance offset prediction accuracy of three methods.

The extreme value of the disturbance force received by the UAV is difficult to measure in a short period of time. It needs to be estimated using a forecast. In a Gaussian distribution-based disturbance force model, it is influenced by the value of σ. By combining traditional weather forecasting and machine learning, we used the UAV’s previous flight data as a dataset and trained the UAV with offset prediction models such as temperature, humidity, atmosphere, wind, wind direction and solar radiation intensity. For the preprocessed samples, tabulation, and label separation, 4/5 (5864) were randomly selected for training the model, and the remaining 1/5 (1466) were used as the test set. The Ridge algorithm of Linear model was selected. Ridge regression is a biased estimation regression method dedicated to collinear data analysis. GaussianNB algorithm of Naive Gaussian Bayesian classifier and XGBClassifier method of XGboost are the Gradient Boosting Decision Tree’s additive model improvement based on the idea of boosting ensemble learning. The predicted results were compared with the original data, and the accuracy rates were 9.424%, 59.426%, and 72.852%, respectively. The results are shown in Figure 4. We selected the model trained by the best XGBClassifier.

According to the prediction result, the discrete degree of risk distribution σ in the disturbance force distribution model was determined by rounding up.

#### 2.2.4. Comprehensive Condition Discrimination Processor

According to the training model, the UAV can fuzzily predict the offset of the UAV after disturbance to determine the safety boundary, which has certain reference significance, but the offset of the fuzzy prediction is not necessarily accurate. If the actual offset does not reach the resilience range threshold adjusted by the UAV, it is fortunate that the UAV only wastes some operational efficiency, and there is no safety risk. Once this offset exceeds the resilience threshold of the UAV’s regulation, then the UAV faces a safety risk. Therefore, we made a comprehensive conditional discrimination processor to determine whether to improve the resilience coefficient ξ according to the discrimination results.

### 2.3. Dynamic Route Planning of Online Real-Time Monitoring Mechanism

Through the design concept of an online real-time monitoring mechanism, the idea of UAV resilience is realized. Based on the combination of the improved A* algorithm and artificial potential fields algorithm, the potential risk compensation penalty weight ω of the UAV 3D flight route planning algorithm is adjusted in real time according to the comprehensive judgments of disturbance force and traction force, so as to realize the real-time variable strategy route planning of UAV.

#### 2.3.1. Track Planning Based on Improved A* Algorithm

**Figure 5:**
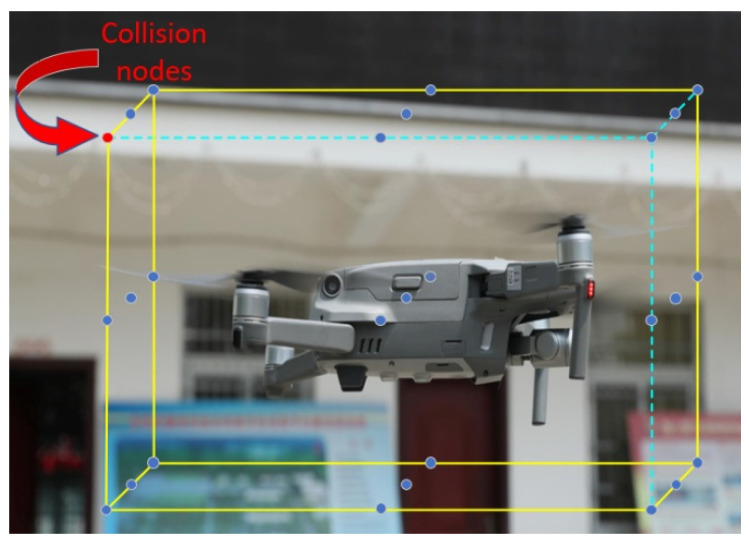
Ring nodes of UAV.

As shown in Figure 5, the 26 nodes around the UAV are the ring nodes, with the UAV as the central point. The ring nodes that precisely cover the width of the UAV’s fuselage are the inner ring nodes, and the ring nodes that are required for the UAV to maintain safe operation in a disturbance scenario are the outer ring nodes. Obviously, the outer ring nodes cover a wider area than the inner ring nodes.

**Figure 6:**
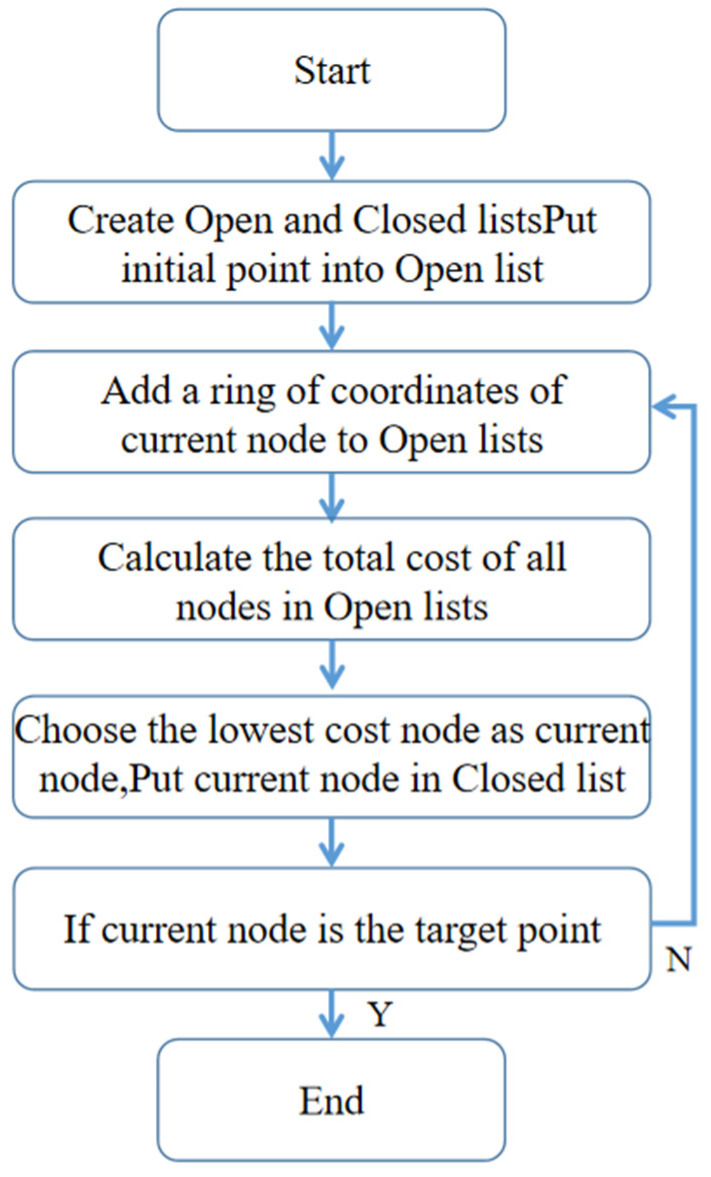
Flow chart of a traditional A* route planning algorithm.

The traditional A* route planning algorithm is simple to calculate and fast to run, which is ideal for use on small UAVs with limited computing power. Its algorithm flow chart is shown in Figure 6.

However, the traditional A* route planning algorithm cannot maintain a safe distance from the obstacle, thus it is improved to meet the requirement of a safe distance.

In order to ensure the safety of the UAV, it is required to maintain the minimum safe distance between the UAV and the environmental obstacles, that is, the safety area $D_{min}$. Let the current environment disturbed with the UAV’s route offset be ξ, and the diameter of the UAV itself be $D_{uav}$, when the minimum safe distance $D_{min}$ fulfills the Formula (7):

(7) $$D_{min}\geq \xi +\frac{1}{2}D_{uav}$$

It can be considered that the UAV maintains a minimum safe distance $D_{min}$ to meet the needs of UAV resilience.

In the improved A* algorithm, the cost of the nodes around the UAV is calculated and sorted through the cost function, and the point with the lowest cost is selected as the next path point. Let the start point be $\left(x_{s},\;y_{s},\;z_{s}\right)$, the target point be $\left(x_{e},\;y_{e},\;z_{e}\right)$, and the UAV currently be at $\left(x_{i},\;y_{i},\;z_{i}\right),$ (s<i<e). This paper uses the Euclidean distance to calculate the estimated cost h(i) of the UAV’s distance from the target point:

(8) $$\{\begin{array}{c} x_{i,e}=x_{i}-x_{e}\\ y_{i,e}=y_{i}-y_{e}\\ z_{i,e}=z_{i}-z_{e} \end{array}$$

(9) $$h\left(i,e\right)=D_{euc}\left(i,e\right)=\sqrt{x_{i,e}{}^{2}+y_{i,e}{}^{2}+z_{i,e}{}^{2}}$$

(10) $$g\left(s,i\right)=\overset{n=i-1}{\underset{n=s}{\sum }}D_{euc}\left(n,n+1\right)$$

g(s,i) represents the cumulative path cost from the starting point to the ith node;n  is an independent variable. In order to maintain a certain distance from obstacles and fully consider the potential safety hazards caused by nearby obstacles to the UAV, the improved A* algorithm modifies the total cost function T(n) in the traditional A* route planning algorithm to make sure that if the UAV does not hit the obstacle, even if it is close to the obstacle, there will be a compensation penalty O(s,i). When the UAV chooses the strategy of bypassing the obstacle as much as possible, it avoids the situation of flying close to the obstacle, so as to realize the UAV’s resilience. The total cost function T(n) of the improved UAV at the current position T(i) is set as follows:

(11) T(i)=g(s,i)+h(i,e)+O(s,i)

(12) $$O\left(s,i\right)=g\left(s,i\right)O_{risk}\omega$$

$O_{risk}\;$ is the potential risk discrimination coefficient. When the UAV’s monitor detects that the outer ring wayfinding point coincides with the obstacle, it is 1, and it is 0 when there is no coincidence. $D_{euc}$ is the Euclidean distance, and ω is the potential risk compensation penalty weight, which is adjusted according to the degree of disturbance. The larger the value, the more safety strategy the UAV will focus on when making route-finding decisions, and the greater the distance reserved for obstacles. On the contrary, it is more focused on the quick strategy, and the reserved distance is smaller. The final total cost function T(i) is organized as follows.

(13) $$T\left(i\right)=\left[\overset{n=i-1}{\underset{n=s}{\sum }}D_{euc}\left(n,n+1\right)\right]\cdot \left(1+O_{risk}\omega \right)+\sqrt{x_{i,e}{}^{2}+y_{i,e}{}^{2}+z_{i,e}{}^{2}}$$

It can be seen that the control risk parameter $O_{risk}$ of the potential risk discrimination coefficient ω is a fixed value set before the start of the task, and it cannot be adjusted at any time during the operation of the UAV. For this reason, we introduce the idea of artificial potential fields algorithm to improve it. By constructing a repulsion function that describes the risk level, the UAV is guided to maintain a safe distance from obstacles.

#### 2.3.2. Improved Resilience Algorithm Based on Artificial Potential Fields Algorithm

**Figure 7:**
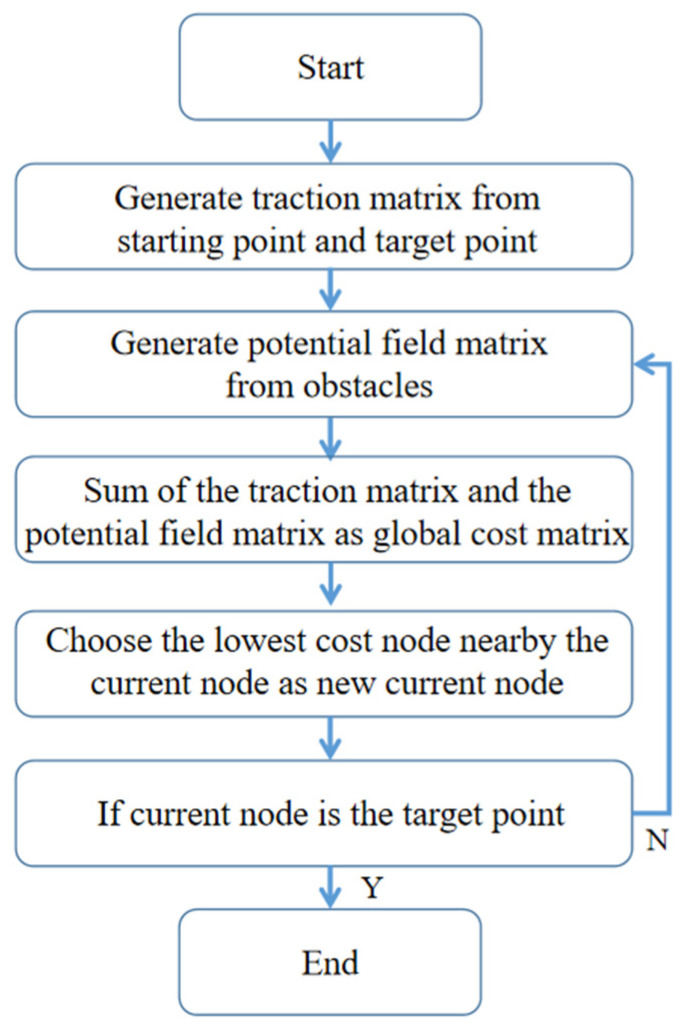
Flow chart of traditional artificial potential field route planning algorithm.

The traditional artificial potential field route planning algorithm is safer compared with the traditional A* algorithm, which reserves a safe distance. However, it is computationally intensive, runs slowly, and tends to fall into local optimization when encountering notch-type obstacles, resulting in the UAV being trapped in the obstacles and wandering indefinitely. The flow chart of the traditional artificial potential field route planning algorithm is shown in Figure 7.

The traditional artificial potential field method is adopted.

The core idea of the artificial potential fields algorithm is to construct the traction function of the distance and the traction force between the UAV and the target point, and the repulsion function of the distance and the repulsion force between the UAV and the obstacle discriminating point. The two functions are compared after weighing the coefficients. In this paper, based on the resilience algorithm, the concepts of traction $F_{tra}$ and repulsion $F_{rep}$ are added to dynamically balance the resilience force. The coordinates of the obstacle node are $\;\left(x_{o},\;y_{o},\;z_{o}\right)$*:*

(14) $$F_{tra}=-\Delta U_{tra}=-k_{tra}\cdot D_{euc}\left(i,e\right)$$

(15) $$F_{rep}=-\Delta U_{rep}=\{\begin{array}{c} k_{rep}\cdot \left(\frac{1}{D\left(i,o\right)}-\frac{1}{R}\right)\cdot {\left(\frac{1}{D\left(i,o\right)}\right)}^{2}\;\left(D\left(i,o\right)\leq D_{min}\right)\\ 0\;\left(D\left(i,o\right)>D_{min}\right) \end{array}\;$$

Once the UAV monitor detects that the obstacle node coincides with the wayfinding point of the UAV outer ring, it means that the obstacle node has entered the UAV risk identification area. The resultant force of traction force $F_{tra}$, repulsion force $F_{rep}$, disturbance force perpendicular to the ground $F_{z}$, disturbance force in the horizontal direction $F_{h}$, and load force G is calculated, where $\vartheta_{F}$ and $\vartheta_{G}$ are the weighting factors of the disturbance force and load. The final resultant force of the UAV is $F_{fin}$.

(16) $$F_{fin}=F_{tra}+F_{rep}+\vartheta_{F}\left(F_{z}+F_{h}\right)+\vartheta_{G}\left(G_{uav}+G_{s}\right)$$

According to the size of the resultant force, whether the UAV is close to the obstacle is determined.

The pseudo code of the REPARE algorithm program is as follows (Algorithm 1),
**Algorithm 1:** Route Planning of Resilience Enhancement.
**Input 1:**  Coordinates of the initial node and the target node1**Input 2:**  Current disturbance field level2**Output:**  List of coordinates of planned route3Choose the appropriate distance ξ according to the disturbance field level4Create Open-list and Close-list5Put the initial node into the Open-list6**Repeat**7**If** Open-list is empty8  Break9**Else** 10  Add a ring of coordinates of the current node to the Open-list 11  Traverse the outer ring coordinates of the current ring node 12  The sensor records the current disturbance force $F_{z}$ $F_{h}$
 13     **If** Crash=Ture (the outer ring nodes meet obstacles) 14    Calculate tractive and repulsive forces $F_{tra}$ $F_{rep}$
 15$\;\;\;\;\;O_{risk}=1$ 16$\;\;\;\;\;\;\;F_{fin}=F_{tra}+F_{rep}+\vartheta_{F}\left(F_{z}+F_{h}\right)+\vartheta_{G}\left(G_{uav}+G_{s}\right)$ 17**    Else** 18$\;\;\;\;\;O_{risk}=0$ 19$\;\;\;\;\;\;\;\;F_{fin}=F_{tra}+\vartheta_{F}\left(F_{z}+F_{h}\right)+\vartheta_{G}\left(G_{uav}+G_{s}\right)$ 20  Calculate the total cost of each node in the inner ring 21Put the current node into the Close-list 22**If** the current node is the target node 23**  Break** 24**If** the current node has adjacent nodes 25  Add adjacent nodes to the Open-list 26**Return** list of nodes planned 27

## 3. Experimental Verifications

### 3.1. Experimental Environment

In order to verify the feasibility of the REPARE algorithm, based on the Python language environment, a simulated environment for UAV operation was built by autonomous coding, and the total range of the map was 100 × 100 × 100 m. Through five buildings of different shapes, a complex urban building complex was simulated. The obstacle location information is shown in Table 1. The authors used the following hardware configuration: Intel Xeon W-2225 4.10 GHz CPU, 32 GB memory. The experiment was run in the Win10 system.

### 3.2. Deployment Algorithms

**Figure 8:**
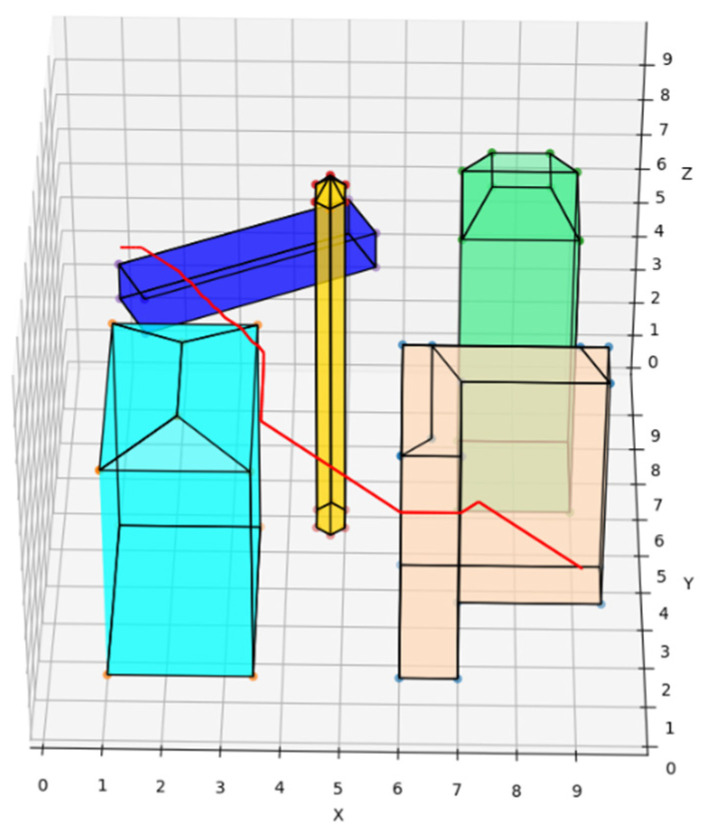
UAV route deploying traditional A* route planning algorithms in disturbance-free scenarios.

**Figure 9:**
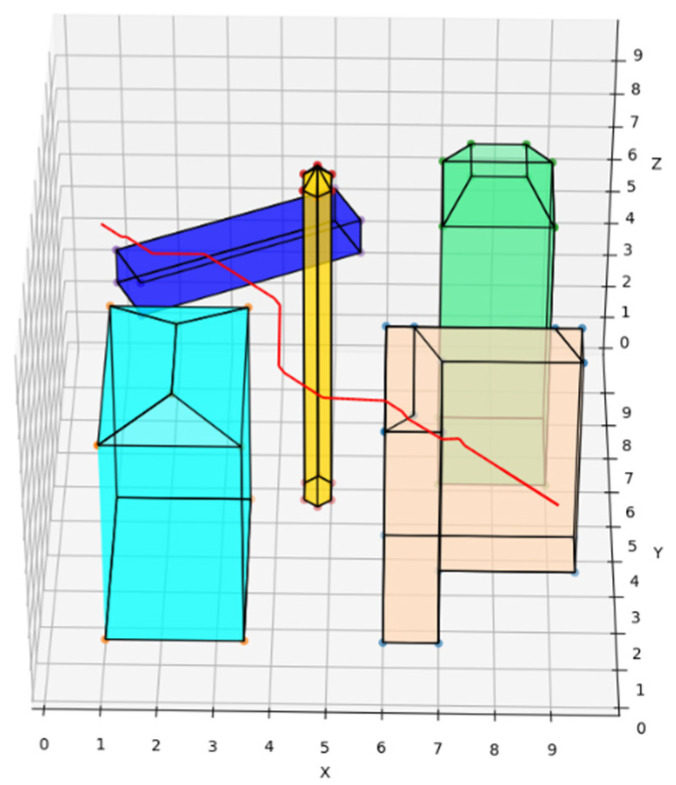
UAV route deploying traditional artificial potential field route planning algorithms in disturbance-free scenarios.

**Figure 10:**
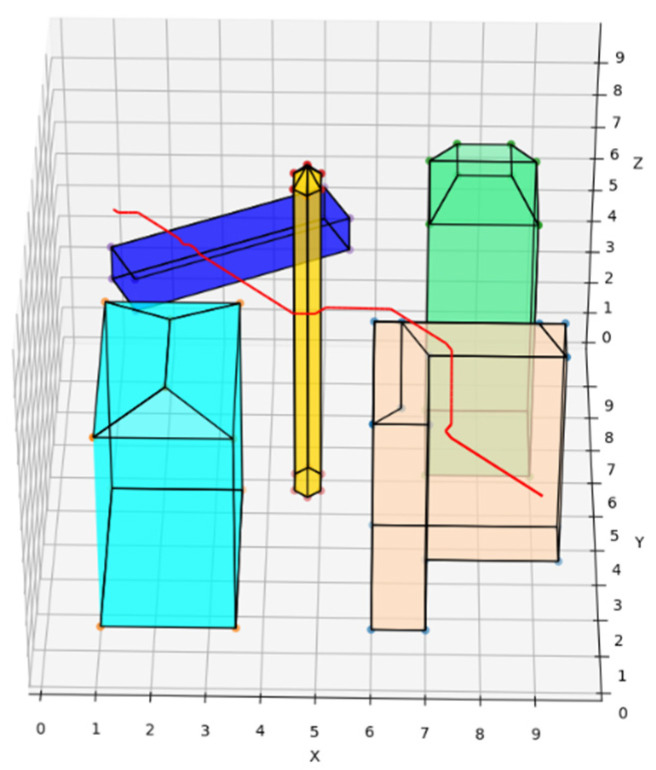
UAV route deploying REPARE algorithm in disturbance-free scenarios.

The traditional A* route planning algorithm, the traditional artificial potential field route planning algorithm, and the REPARE algorithm are deployed in the disturbance-free scenario, as shown in Figure 8, Figure 9 and Figure 10. The red line represents the route that the UAV finds autonomously deploying the route planning algorithms. Comparing the routes of the three algorithms, one is to find that the UAV routes deployed with the traditional A* route planning algorithm and REPARE algorithm are similar in distance, and the UAV routes deployed with the traditional artificial potential field route planning algorithm are farther away. In the disturbance-free scenario, the UAV deployed with REPARE algorithm reduces the work intensity of the obstacle avoidance module, and a more reasonable and efficient route can be planned.

The starting and target points are randomly generated, and after a large number of repeated experiments, the average running time and distance of the three algorithms are compared, and they are normalized by the parameters of the traditional artificial potential field route planning algorithm. The comparison results are shown in Table 2.

**Figure 11:**
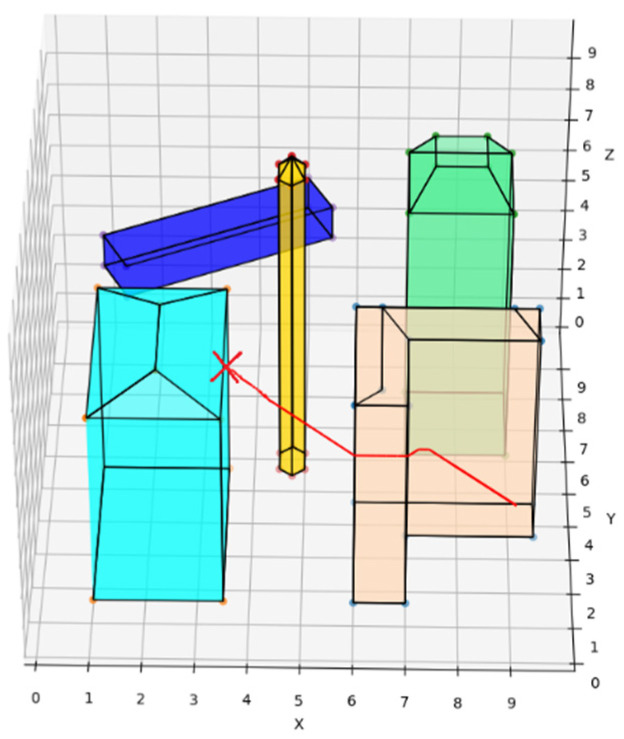
UAV route deploying traditional A* route planning algorithms in disturbance scenarios.

The REPARE algorithm is deployed in the environment. Such intrinsic parameters such as UAV deadweight and load are pre-set. The external disturbance parameters are wind, rain, magnetic field, etc. Subjecting the UAV to disturbance forces under different loads creates a tendency to deviate from the course. The trend is corrected by the REPARE algorithm according to the local conditions. Compared to the UAVs deployed with the traditional A* route planning algorithm and the traditional artificial potential field route planning algorithm as a control group, the UAVs deployed with the traditional A* algorithm do not reserve a safe distance and will collide once they are disturbed. A crash is shown as the red “X” in Figure 11. The UAVs deploying the traditional artificial potential field route planning algorithm and the REPARE algorithm have the ability to resist disturbance. Among them, the REPARE algorithm leaves a smaller safety margin because the algorithm identifies less intensity of disturbance, which can reduce the safety requirements and improve the efficiency of the UAV operation.

**Figure 12:**
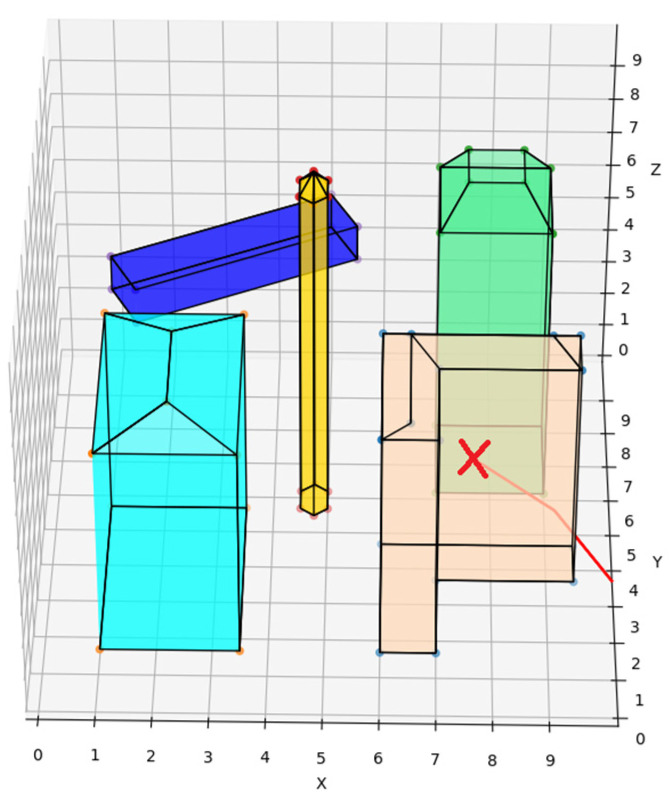
Deployment of local optimization of traditional artificial potential field route planning algorithms.

**Figure 13:**
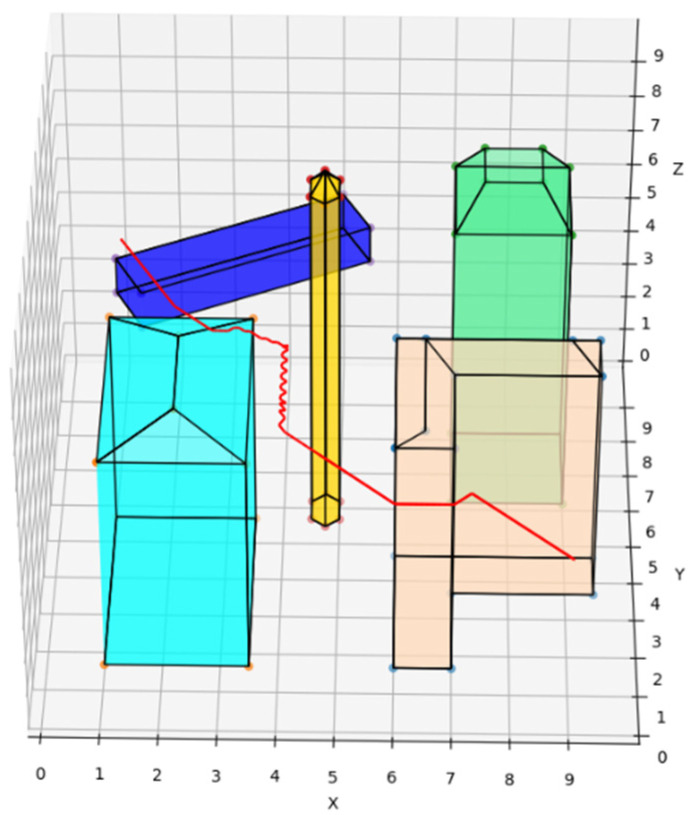
UAV route deploying traditional artificial potential field route planning algorithms in disturbance scenarios.

**Figure 14:**
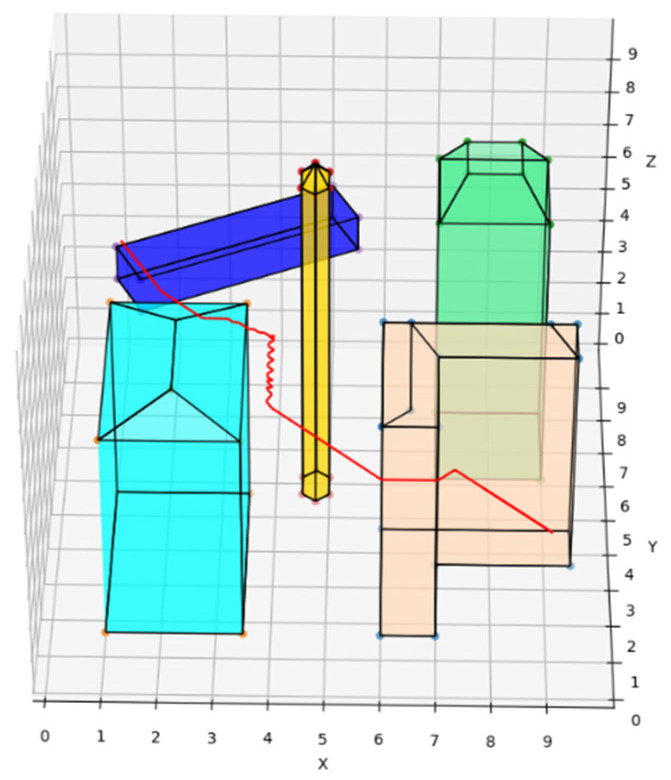
UAV route deploying REPARE algorithm in disturbance scenarios.

In particular, when the UAV deploying the traditional artificial potential field route planning algorithm is too close to a notch-type obstacle (peach puff), a local optimization problem arises, and the UAV trembles infinitely in the notch at the spot represented by the red ”X” and cannot avoid the obstacle, as is shown in Figure 12. The REPARE algorithm can avoid local optimization problems by introducing the Open-list of the A* algorithm. The routes of the UAV deploying the traditional artificial potential field route planning algorithm and the REPARE algorithm in the disturbance scenario are shown in Figure 13 and Figure 14.

### 3.3. Risk Assessment of UAVs

The larger the actual load of the UAV is, the more kinetic energy the UAV has to be paid when adjusting the sailing speed, altitude and attitude. To take a civil light quadrotor UAV as an example, according to the kinetic energy classification, the load and disturbance wind speed can be obtained as follows in Table 3. It should be noted that:

1. The level of “no disturbance” means no wind conditions, not absolutely no wind, but the wind speed is low, which has little impact on the UAV. It only needs to be considered in the case of an extreme load of the UAV.

2. Under severe disturbance, it is possible that the equivalent mass of the disturbance force in the z-axis direction (heavy rain) exceeds the maximum load of the UAV. Thus, the flying stops.

We selected three representative route results to demonstrate the UAV resilience decision under different loads and different risks.

**Figure 15:**
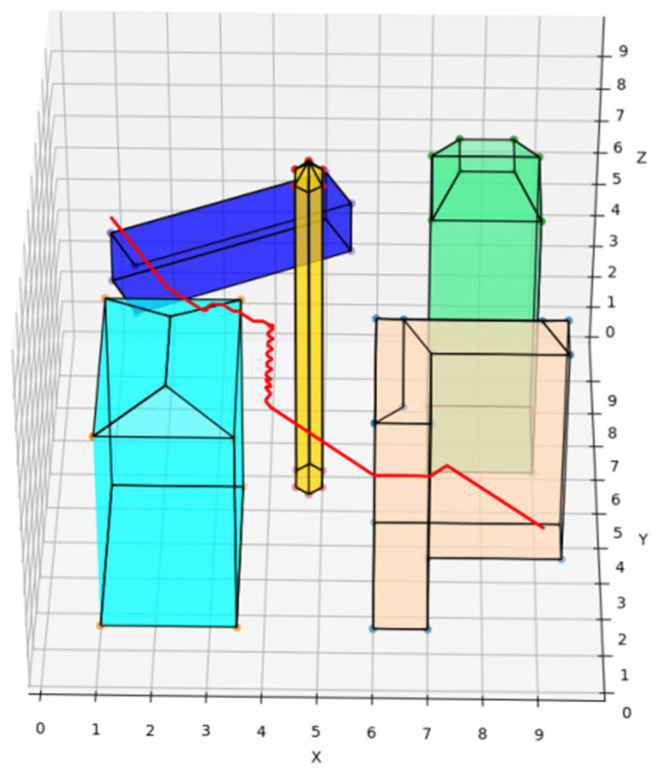
UAV route with light load and mild disturbance.

As shown in Figure 15, in the case of mild disturbance and light load, it can be seen that the UAV has a certain tremor when it is disturbed, can respond in time, hardly deviates from the route, and runs fast.

**Figure 16:**
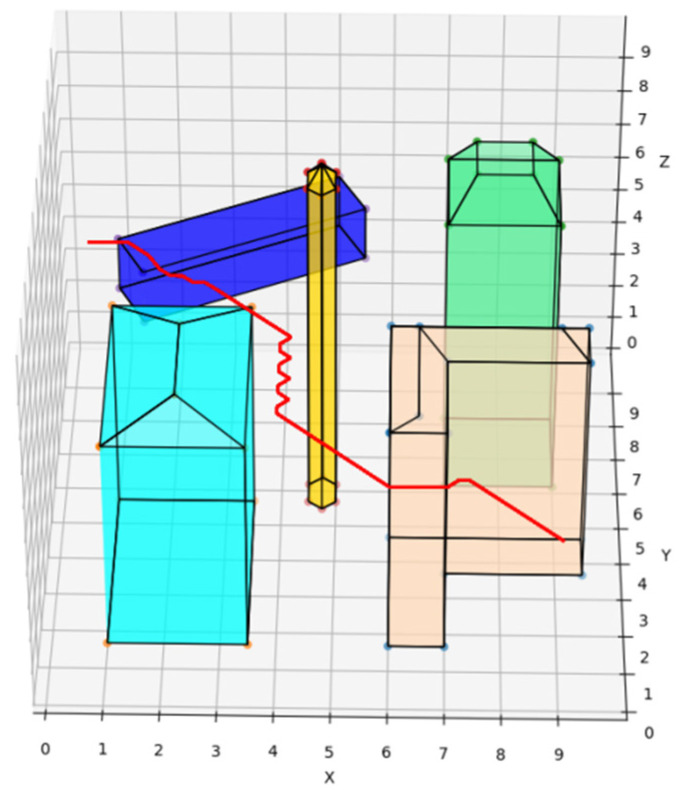
UAV route with heavy load and mild disturbance.

As shown in Figure 16, in the case of mild disturbance and heavy load, the load of the UAV has almost reached the limit of the driving force of the UAV; the response speed is obviously slow, the running speed is reduced, the route correction time increases, but the distance from the obstacle still remains at the same safe distance, and the running speed is slow.

**Figure 17:**
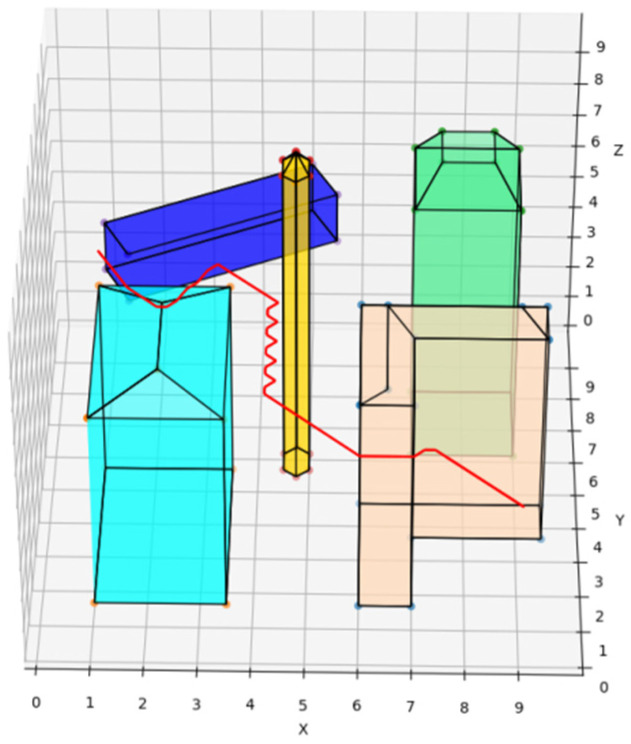
UAV route with light load and heavy disturbance.

As shown in Figure 17, in the case of heavy disturbance and light load, the UAV needs more driving force to counteract the disturbance. Compared with the case of heavy load and mild disturbance, the operating speed is increased to counteract the disturbance. At the same time, a larger safety margin is left, and the running speed is moderate.

A comparison of the minimum safe distances of UAVs deployed with the REPARE algorithm for different disturbance scenarios is shown in Table 4.

### 3.4. Monte Carlo Simulation Verification

In the simulated environment, after a random disturbance was generated using the Monte Carlo method, the offset of the UAV was recorded and classified: an offset perpendicular to the ground direction $\xi_{ver}$, and an offset parallel to the ground direction $\xi_{hor}$.

According to this theory, we extended the independent variable to two dimensions. The offset perpendicular to the ground $\xi_{ver}$ and the offset parallel to the ground $\xi_{hor}$ were used as independent variables, and the probability $P_{joint}$ of occurrence was the dependent variable.

A two-dimensional distribution was obtained:

(17) $$f\left(\xi_{\mathrm{ver}},\xi_{\mathrm{hor}}\right)={\left(2{\pi \sigma }_{1}\sigma_{2}\sqrt{1-\rho^{2}}\right)}^{-1}\exp\left[-\frac{1}{2\left(1-\rho^{2}\right)}\left[\frac{{\left(\xi_{\mathrm{ver}}-\mu_{1}\right)}^{2}}{\sigma_{1}{}^{2}}-\frac{2\rho \left(\xi_{\mathrm{ver}}-\mu_{1}\right)\left(\xi_{\mathrm{hor}}-\mu_{2}\right)}{\sigma_{1}\sigma_{2}}+\frac{{\left(\xi_{\mathrm{hor}}-\mu_{2}\right)}^{2}}{\sigma_{2}{}^{2}}\right]\right]\;$$

**Figure 18:**
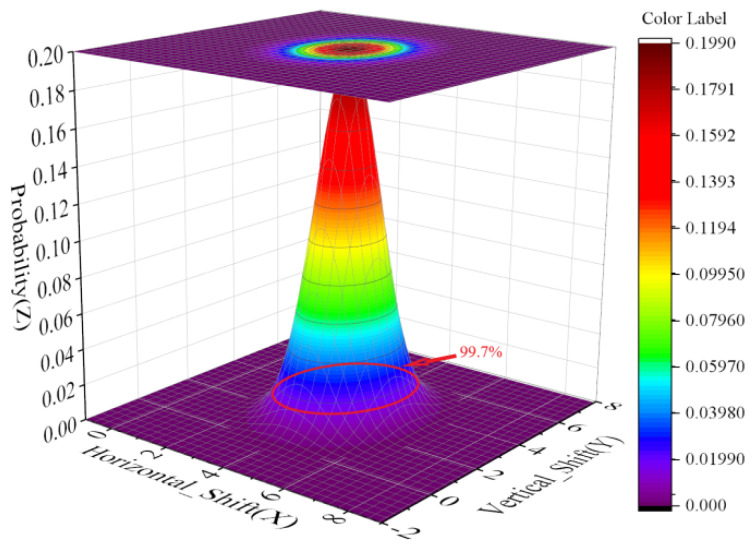
“Offset-probability” heat map of UAV route planning.

A three-dimensional coordinate system was established to form an “offset-probability” heat map. As shown in Figure 18, the safety domain determined according to the REPARE algorithm is drawn according to the disturbance offset predicted in this paper at the place marked by the red circle, which meets the requirements of the Pauta criterion. Therefore, we think that the UAV resilience adjustment 3D route planning algorithm based on risk assessment and artificial potential fields optimization designed in this paper is safe and reliable.

## 4. Conclusions

In order to solve the real-time route safety planning problem of the rotor UAV in the disturbed complex three-dimensional scene, this paper was based on the traditional A* algorithm and the artificial potential fields algorithm and was combined with such concepts as the resilience system, the mechanism of determining a reasonable safety range, and the mechanism of online real-time monitoring. In this paper, a resilience enhancement algorithm REPARE for the 3D route planning of rotor UAVs suitable for flying in a complex urban environment of buildings was proposed. Taking a high-risk scene operation in wind and rain as an example, the simulation and calculational analysis of the whole process of resilient route planning were carried out. The experimental results showed that in the disturbed complex building environment, the UAV deployed with the REPARE algorithm is better than the traditional algorithm. It is more efficient and safer, and it enhances the resilience of the UAV.

The present algorithm also has limitations. When the map accuracy was moderate, in a 100 × 100 × 100 m map, whose minimum map unit was 1 m, the global operation time was low. If the accuracy of the map increased, the amount of operations would increase significantly. Future work can continue to optimize the algorithm to calculate the route in steps as needed for flights during UAV operation.

## Figures and Tables

**Table 1:** Distribution of obstacles.

No.	Coordinate of Nodes	Color
1	(70,15,0), (60,15,0), (60,45,0), (70,15,65), (95,35,0), (70,35,0), (60,15,65), (60,45,65), (95,45,0), (95,45,65), (95,35,65), (70,35,65), (65,15,70), (65,40,70), (90,40,70)	Peach puff
2	(10,15,0), (10,55,0), (35,55,0), (35,15,0), (10,15,60), (10,55,60), (35,55,60), (35,15,60), (22,25,65), (22,45,65)	Cyan
3	(70,60,0), (70,80,0), (90,80,0), (90,60,0), (70,60,80), (70,80,80), (90,80,80), (90,60,80), (75,65,90), (85,65,90), (85,75,90), (75,75,90)	Green
4	(15,65,45), (10,75,45), (50,95,45), (55,85,45), (15,65,60), (10,75,60), (50,95,60), (55,85,60)	Blue
5	(45,55,0), (48,53,0), (50,55,0), (50,60,0), (48,62,0), (45,60,0), (45,55,95), (48,53,95), (50,55,95), (50,60,95), (48,62,95), (45,60,95), (48,58,99)	Yellow

**Table 2:** Comparison of the three algorithms’ average time and distance.

Algorithm	Route Distance	Time
Traditional A* route planning algorithm	0.952	0.558
Traditional artificial potential field route planning algorithm	1	1
REPARE	0.973	0.655

**Table 3:** Schematic representation of the degree of adjustment of the resilience system.

Load/Disturbance	Dead Load	Light Load	Moderate Load	Heavy Load
No disturbance	Resilience system shuts down	Resilience system shuts down	Lower resilience factor	Intermediate resilience factor
Mild disturbance	Lower resilience factor	Lower resilience factor	Intermediate resilience factor	Advanced resilience factor
Moderate disturbance	Intermediate resilience factor	Intermediate resilience factor	Advanced resilience factor	Advanced resilience factor
Heavy disturbance	Intermediate resilience factor	Advanced resilience factor	Grounded	Grounded

**Table 4:** Comparison of minimum safe distance under different disturbance scenarios.

Condition	Resilience Factor	Minimum Safe Distance
light load and mild disturbance	Intermediate	3.5
heavy load and mild disturbance	Advanced	4.5
light load and heavy disturbance	Advanced	5.5

## Data Availability

Not applicable.

## Appendix A

**Table A1:** Symbols that appear in this article.

Symbol	Meaning
$F_{r}$	vertical force of the rain
$P_{r}$	pressure by raindrops
$S_{r}$	effective area of the UAV’s body
$H_{r}$	amount of precipitation
$\rho_{r}$	density of the rainwater
$V_{r}$	speed of the raindrops
$\eta_{r}$	momentum loss of rainwater
t	rain’s unit of time
$F_{zMax}$	max driving force of the UAV along the Z-axis
$\delta_{r}$	resilience coefficient fulfilling the flight safety
$G_{uav}$	UAV self-weight
$G_{s}$	UAV load
$\vec{V_{\mu F}}$	forecasted average wind speed
$\vec{V_{MaxF}}$	maximum wind speed
$t_{w}$	wind’s unit of time
$\vec{V_{\mu S}}$	current wind speed measured by UAV
$V_{res}$	speed of resilience
$\vec{V_{d}}$	mission required speed
$\vec{V_{uav}}$	maximum speed that the UAV can reach in a no-wind
$\vec{v}$	UAV’s flight speed relative to the ground
$F_{hmax}$	max force in the horizontal direction of the UAV
C	air resistance coefficient
$\rho_{a}$	air density
$S_{w}$	equivalent area of the UAV in contact with the wind
σ	level of risk
μ	level of disturbance
n(x)	probability density function of the one-dimensional shift
$D_{min}$	minimum safe distance of UAV
$D_{uav}$	diameter of the UAV
ξ	UAV’s route offset
h(i,e)	estimated cost of the UAV’s distance from the target point
g(s,i)	cumulative path cost from the starting point to the *i*th node
O(s,i)	compensation penalty
T(n)	total cost function
$F_{tra}$	traction force
$F_{rep}$	repulsion force
$\vartheta_{F}$	weighting factors of disturbance
$\vartheta_{G}$	weighting factors of load
$F_{fin}$	final resultant force of the UAV
